# Negative Association Between Smoking and Positive SARS-CoV-2 Testing: Results From a Swiss Outpatient Sample Population

**DOI:** 10.3389/fpubh.2021.731981

**Published:** 2021-11-05

**Authors:** Juan R. Vallarta-Robledo, José Luis Sandoval, Stéphanie Baggio, Julien Salamun, Frédérique Jacquérioz, Hervé Spechbach, Idris Guessous

**Affiliations:** ^1^Faculty of Medicine, University of Geneva, Geneva, Switzerland; ^2^Division and Department of Primary Care Medicine, Geneva University Hospitals, Geneva, Switzerland; ^3^Department of Oncology, Geneva University Hospitals, Geneva, Switzerland; ^4^Division of Prison Health, Geneva University Hospitals, Geneva, Switzerland; ^5^Department of Justice and Home Affairs of the Canton of Zurich, Office of Corrections, Zurich, Switzerland

**Keywords:** SARS-CoV-2, COVID-19, outpatient testing, smoking, public health

## Abstract

To date, most of the evidence suggests that smoking is negatively associated with testing positive for SARS-CoV-2. However, evidence has several methodological limitations. Using an outpatient sample population, we analyzed the association of testing positive for SARS-CoV-2 and smoking considering comorbidities, socioeconomic and demographic factors. Baseline data were obtained from a cohort during the first wave of the pandemic in Geneva, Switzerland (March-April 2020). RT-PCR tests were carried out on individuals suspected of having SARS-CoV-2 according to the testing strategy at that time. Logistic regressions were performed to test the association of smoking and testing positive for SARS-CoV-2 and further adjusted for comorbidities, socioeconomic and demographic factors. The sample included 5,169 participants; 60% were women and the mean age was 41 years. The unadjusted OR for testing positive for SARS-CoV-2 was 0.46 (CI: 0.38–0.54). After adjustment for comorbidities, socioeconomic and demographic factors, smoking was still negatively associated with testing positive for SARS-CoV-2 (OR: 0.44; CI: 0.35–0.77). Women (OR: 0.79; CI: 0.69–0.91), higher postal income (OR: 0.97; CI: 0.95–0.99), having respiratory (OR: 0.68; CI: 0.55–0.84) and immunosuppressive disorders (OR: 0.63; CI: 0.44–0.88) also showed independent negative associations with a positive test for SARS-CoV-2. Smoking was negatively associated with a positive test for SARS-CoV-2 independently of comorbidities, socioeconomic and demographic factors. Since having respiratory or immunosuppressive conditions and being females and healthcare workers were similarly negatively associated with SARS-CoV-2 positive testing, we hypothesize that risk factor-related protective or testing behaviors could have induced a negative association with SARS-CoV-2.

## Introduction

Smoking is considered a risk factor for respiratory infectious diseases (1). Hence, it could be hypothesized that smokers are also at an increased risk of SARS-CoV-2 infection. However, much of the evidence shows that smoking is negatively associated with COVID-19 and its complications, and to date, there is still an ongoing debate about the role smoking plays in the infection risk for SARS-CoV-2 (2).

On one hand, some authors attribute a protective effect due to nicotine and its interaction with the immune and renin-angiotensin systems (3). On the contrary, other studies suggest that this negative association could be due to methodological bias; with most of the studies being from hospital settings and having underrepresented smoking prevalence (4, 5). Furthermore, data are usually not adjusted for potential confounding factors, such as comorbidities, age, sex, and socioeconomic status, which could help to better understand this association (4). Furthermore, populations concerned for their owns health (6), as we believe could be individuals at higher risk for COVID-19 severity (i.e. individuals with comorbidities and smokers), seems to take more protective measures against COVID-19, and therefore, this may be a possible explanation for the negative association between smoking and testing positive for SARS-CoV-2 infection (7) although contradictory findings have been reported (8).

Only a few studies have assessed the association of smoking and testing positive for SARS-CoV-2 at the population level with further adjustment for comorbidities and socioeconomic and demographic factors (7–10). Results are contradictory, with some studies reporting a negative association (7, 9), while others showing positive (8), or no association (10).

Because of this lack of agreement, and the dangerous implications falsely claiming of a protective effect of smoking for SARS-CoV-2 infection could have, we aimed to explore the association of smoking and the probability of testing positive for SARS-CoV-2 in a sample population with smoking prevalence being similar to the national statistics while adjusting for comorbidities and socioeconomic and demographic data.

## Methods

### Population Data

Baseline data were collected from a cohort study of outpatients attending testing centers for SARS-CoV-2 in the Geneva University Hospitals (HUG) from March 11 to April 21 of 2020, corresponding to the first wave of the pandemic in Geneva, Switzerland. Information was collected by trained personal using a standardized questionnaire (Supplementary Appendix 1).

Testing for SARS-CoV-2 was carried out according to the federal recommendations at the time: individuals with any respiratory symptom, fever, risk factors for COVID-19 complications (age >65 years, hypertension, chronic respiratory diseases, diabetes, heart diseases, cancer, and immunosuppressive conditions), close contact with a COVID-19 positive individual, and/or a recent trip (previous 14 days) to a region known for having a high incidence of COVID-19. Additionally, healthcare workers were tested if suspected of a SARS-CoV-2 infection and if they had close contact with individuals at risk of COVID-19 complications.

Individuals below 16 years, non-residents of the state of Geneva, without a valid RT-PCR result, or who refused the use of their data for research were excluded. The study was approved by the Cantonal Ethics Research Committee of Geneva (2020-00813).

### COVID-19 Diagnosis and Covariates

SARS-CoV-2 was measured using a reverse transcriptase–polymerase chain reaction (RT-PCR) test in naso-oropharyngeal swabs. The tests were carried out by nurses or medical doctors following standardized procedures at the HUG virology laboratory (the Swiss national reference laboratory for SARS-CoV-2) and performed based on manufacturer's instructions, which initially included eMAG (bioMérieux, Marcy l'Etoile, France) and the Charité/Berlin RT-PCR protocol and were followed by the BD SARS-CoV-2 kit for BD Max system (Becton Dickinson & Co, Franklin Lakes, NJ, USA) and Cobas 6800 SARS CoV2 RT-PCR (Roche Diagnostics, Rotkreuz, Switzerland). Diagnosis for SARS-CoV-2 infection was either positive or undetected. Non-hospitalized SARS-CoV-2 positive patients were isolated at home for 10 days and followed-up by the local health authorities via phone calls. Additional medical evaluations were carried out for those presenting symptoms of disease progression. However, such information was not available for this study.

Age (years), gender, cardiovascular diseases and related risk factors (hypertension and diabetes), immunosuppressive conditions (including cancer), chronic respiratory diseases, a recent trip to areas at risk of COVID-19, and closeness to a COVID-19 individual were self-reported and considered as covariates. To account for economic status, we obtained the annual postal household income of married couples (1 CHF = 1.12 USD, in January of 2021) for the year 2018 reported by the Cantonal Office of Statistics (COS, www.ge.ch/statistique) and assigned to the postal place of residence of each participant. The COS reports income only on married individuals, as the information for single subjects may be biased due to such a population is more likely to share a household.

### Statistical Analysis

Continuous data are described as mean and standard deviation (±SD), and categorical information as frequencies and percentages (%). We performed simple logistic regression models to assess the association between SARS-CoV-2 positive testing and smoking and related covariates. We used multiple logistic regression to assess the association of smoking and testing positive for SARS-CoV-2 infection adjusting for comorbidities, socioeconomic and demographic covariates. Results from the regression analyses are described as Odd Ratios (OR) and 95% confidence intervals (CI).

As sensitivity analyses, we performed multiple logistic regressions stratified by gender and non-healthcare workers. Analyses were performed in R 3.6.3.

## Results

The initial dataset contained 7,651 participants of whom 5,349 were further included in the study after removing non-residents of the state of Geneva and individuals without a valid RT-PCR result. There were 180 (3%) participants with missing data in covariates and assumed to be missing completely at random (Supplementary Table 1, Supplementary Materials). Thus, the final study sample included 5,169 participants. From this sample, 60% were women, the mean age was 41.0 ± 13.7 and the smoking prevalence was 24% (Table 1).

**Table 1:** Population characteristics (overall and stratified by SARS-CoV-2 test results).

	**ALL**	**Undetected SARS-CoV-2**	**Positive SARS-CoV-2**	***p*-value^a^**
N	5,169	3,973 (77%)	1,196 (23%)	-
Smokers	1,246 (24%)	1,073 (27%)	173 (15%)	<0.001
Age (years)	41.0 ± 13.7	40.9 ± 13.7	41.3 ± 13.8	0.37
Women	3,093 (60%)	2,429 (61%)	664 (56%)	<0.001
Healthcare workers	2,023 (39%)	1,623 (41%)	400 (33%)	<0.001
Household postal income (USD)	162,419 ± 35,488	162,981 ± 31,834	160,549 ± 31,145	0.03
Respiratory diseases	676 (13%)	558 (14%)	118 (10%)	<0.001
Cardiovascular diseases and risk factors	659 (13%)	520 (13%)	139 (12%)	0.20
Immunosuppressive conditions	266 (5%)	223 (6%)	43 (4%)	<0.001
Trip to a COVID-19 risk area	443 (9%)	323 (8%)	120 (10%)	0.04
Contact with a COVID-19 positive individual	2,098 (41%)	1,544 (39%)	554 (46%)	<0.001

a *Undetected SARS-CoV-2 vs. positive SARS-CoV-2 test using student t tests for numeric data an chi-square tests for categorical information*.

Unadjusted models showed that smokers (OR: 0.46, CI: 0.38–0.54), women (OR: 0.79, CI: 0.70–0.90), postal household income (OR: 0.98, CI: 0.96–0.99), healthcare workers (OR: 0.73, CI: 0.63–0.83), and individuals with respiratory (OR: 0.67, CI: 0.54–0.82) and immunosuppressive conditions (OR: 0.63, CI: 0.44–0.86) were less likely to test positive for COVID-19 (Table 2). On contrary, participants traveling to areas with a high incidence of COVID-19 (OR: 1.26, CI: 1.01–1.57) and that had close contact with a SARS-Cov-2 infected individual (OR: 1.36, CI: 1.19–1.55) were at higher risk of having a positive test. The risk factors of age and cardiovascular diseases were not statistically associated with a positive SARS-CoV-2 test.

**Table 2:** Association of smoking and positive testing for SARS-CoV-2 and potential related factors.

	**ALL, Unadjusted**		**ALL, Adjusted**	
	**OR (95% CI)**	***p*-value**	**OR (95% CI)**	***p*-value**
Smokers (yes vs. no)	0.46 (0.38, 0.54)	<0.001	0.44 (0.35, 0.77)	<0.001
Age (years)	1.00 (0.99, 1.00)	0.37	1.00 (0.99, 1.00)	0.12
Women vs. men	0.79 (0.70, 0.90)	<0.001	0.79 (0.69, 0.91)	<0.001
Healthcare workers vs. general population	0.73 (0.63, 0.83)	<0.001	0.61 (0.52, 0.71)	<0.001
Household postal income (per 10,000 USD)	0.98 (0.96, 0.99)	0.04	0.97 (0.95, 0.99)	0.02
Respiratory diseases (yes vs. no)	0.67 (0.54, 0.82)	<0.001	0.68 (0.55, 0.84)	0.001
Cardiovascular diseases and risk factors (yes vs. no)	0.87 (0.71, 1.06)	0.18	0.82 (0.66, 1.01)	0.07
Immunosuppressive conditions (yes vs. no)	0.63 (0.44, 0.86)	0.006	0.63 (0.44, 0.88)	0.009
Trip to a COVID-19 risk area (yes vs. no)	1.26 (1.01, 1.57)	0.04	1.32 (1.05, 1.65)	0.02
Contact with a SARS-CoV-2 positive individual (yes vs. no)	1.36 (1.19, 1.55)	<0.001	1.61 (1.39, 1.85)	<0.001

*Analyses are shown unadjusted and adjusted (comorbidities, socioeconomic and demographic factors) for the entire studied population (*n* = 5,169)*.

The model adjusted for the covariates showed similar results. Smokers (OR: 0.44, CI: 0.35–0.77), women (OR: 0.79, CI: 0.69–0.91), postal household income (OR: 0.97, CI: 0.95–0.99), healthcare workers (OR: 0.61, CI: 0.52, 0.71), and individuals with respiratory (OR: 0.68, CI: 0.55–0.84) and immunosuppressive conditions (OR: 0.63, CI: 0.44–0.88) presented lower probability of testing positive for SARS-CoV-2 while traveling to an area with a high incidence of COVID-19 (OR: 1.32, CI: 1.05–1.65) and being in close contact with a SARS-CoV-2 positive individual increased the probability of a positive test result (OR: 1.61, CI: 1.39–1.85).

Adjusted multiple logistic regressions stratified by gender and restricted to non-healthcare workers (Supplementary Table 2, Supplementary Materials) yielded similar results to the previous model. Smoking was negatively associated with testing positive for SARS-CoV-2 in non-healthcare workers (OR: 0.41, CI: 0.33–0.51), and men and women (OR: 0.43, CI: 0.33–0.56 and OR: 0.44, CI: 0.34–0.57, respectively).

## Discussion

Using outpatient sample data, we found that smoking was negatively associated with testing positive for SARS-CoV-2. This negative association remained after adjustment for comorbidities, socioeconomic and demographic factors. Analyses restricted to non-healthcare workers, men and women showed the same negative association. Contrary to most of the studies (4, 5), the smoking prevalence in our data (24%) was similar to the national statistics (27%) (11).

Like most of the studies evaluating the association of smoking and testing positive for SARS-CoV-2, after further adjustment for comorbidities, socioeconomic and demographic factors at the community level (7, 9), we found a negative association of smoking and testing positive for SARS-CoV-2. While the same negative association was also observed for healthcare workers, it is worth mentioning that in these individuals the testing strategy was more extensive and would explain the negative association between healthcare workers and a positive SARS-CoV-2 test. Additionally, we also found a negative association with a history of respiratory and immunosuppressive conditions (7). Such results may suggest that populations at higher risk of COVID-19 complications, or worried about their health status, took greater protective measures against SARS-CoV-2 infection (6, 7). Jackson et al. (8) found that despite smokers being more concerned about being infected with SARS-CoV-2, they had lower adherence to health recommendations in the United Kingdom. However, no negative association of smoking was found in this study, which may indicate behavioral differences across populations, and positive SARS-CoV-2 infection was self-reported.

Other authors have suggested that this negative association of smoking and COVID-19 could be due to either an increase of the testing rates, as these populations are usually at a higher priority for SARS-CoV-2 testing (5, 9), or an underrepresentation, as they may have lower access to healthcare or die before being tested (5, 8). However, smoking prevalence in our study was similar to the national statistics suggesting the negative association was not due to misrepresentation of this population. Furthermore, analyses were adjusted for household income. As such, socioeconomic status does not seem to explain the negative association between smoking and SARS-CoV-2 positive testing. Another hypothesis is based on the idea that alterations in the nasopharyngeal viral load in smokers may reduce the sensitivity of the RT-PCR tests (9). Although this hypothesis could explain why smokers may be associated with higher false-negatives rates for SARS-CoV-2 RT-PCR testing (12), it would not explain lower SARS-CoV-2 antibody prevalence in smokers as found in other populations (13), Additionally, smokers are more prone to develop chronic pulmonary disease, and therefore, to use inhaled glucocorticoids, which use, has been suggested to reduce the replication of SARS-CoV-2 (14), In our study, only 15% of smokers reported having respiratory diseases which would not explain, at least entirely, the negative association of SARS-CoV-2 in smokers. While possible protective biological pathways related to nicotine (but not smoking) have been theoretically described (3), further studies are required to test and validate them.

Like previous studies (7–10), we found a positive association of testing positive for SARS-CoV-2 with older age (only in models stratified for men and the non-healthcare population, Supplementary Table 2), being male, socioeconomically disadvantaged groups, and having a known exposure to COVID-19. Associations between age and COVID-19 have been attributed to a weaker immune system (15), while gender discrepancies have been related to biological and psychosocial determinants (16). Furthermore, deprived individuals are at higher risk of SARS-CoV-2 infection since they face unequal access to health, higher exposure to infection risk factors (17), and their living environments facilitate the persistence of clusters of infection/transmission (18).

This study has various strengths. We had access to the results of RT-PCR tests to diagnose COVID-19, known for their high specificity (19), and we did not rely on self-reports of positive tests for SARS-CoV-2 infection (8). Furthermore, our sample had a smoking prevalence similar to the national prevalence, and we were able to adjust for various comorbidities, socioeconomic and demographic factors which allowed us to control for determinants that may contribute to the negative association of smoking and testing positive for SARS-CoV-2 infection. Also, we tested the robustness of our findings in different subpopulations (non-healthcare workers, women, and men) and observed similar results.

Yet, our study has several limitations. This was an observational study using baseline data; thus, causality cannot be inferred. Data were not collected using a random sampling procedure, but rather a restrictive and changing approach where women (60%) and healthcare workers (39%) were overrepresented. This may cause selection and collider bias (20) as individuals at higher risk may be more likely to be tested. Additionally, data were obtained during the beginning of the pandemic and in dedicated outpatient centers of the HUG which may not be entirely representative of the population of Geneva and subsequent waves of the COVID-19 pandemic. We used the socioeconomic status at the postal level which limits our findings at an aggregated scale. Likewise, we were not able to identify former smokers or adjust our analysis for pack-year consumption levels because this information was not collected in the survey. Furthermore, smoking status and concomitant diseases were self-reported which may cause over-underestimation issues due to social desirability or misunderstanding of the condition. Finally, we lacked adjusting the analysis to the prognosis of the disease in those who became infected with the virus.

Due to the above limitations, our results should be taken cautiously, and smoking cessation should be encouraged and prioritized due to smoking is a well-known risk factor for several diseases, including respiratory infections (21). Indeed, there is wide evidence indicating that smokers are at higher risk of facing worst disease progression and death following a SARS-CoV-2 infection (22, 23).

## Conclusions

Smoking was negatively associated with testing positive for SARS-CoV-2 infection independently of comorbidities and socioeconomic and demographic factors in a population with a smoking prevalence similar to the national statistics. Current pathways of how smoking is related to SARS-CoV-2 infection are still unclear, and evidence and quality of such association are limited and subject to bias. As a history of respiratory or immunosuppressive conditions, and being females and healthcare workers were also negatively associated with testing positive for SARS-CoV-2, we hypothesize that risk factor-related protective or testing behaviors (selection and collider bias) could have induced a negative association with SARS-CoV-2 during the first wave of the COVID-19 pandemic. This should be seriously considered before claiming a protective effect of smoking for SARS-CoV-2 infection, especially since the evidence shows that if smokers develop COVID-19, they have an increased risk of severe health complications. We encourage further population-based studies that validate our assumptions by exploring the association of smoking and SARS-CoV-2 infection considering cultural and behavioral factors, as well as levels of adherence to recommendations from health authorities during the pandemic.

## Data Availability Statement

The raw data supporting the conclusions of this article will be made available by the authors, without undue reservation.

## Ethics Statement

The studies involving human participants were reviewed and approved by Cantonal Ethics Research Committee of Geneva. Written informed consent to participate in this study was provided by the participants' legal guardian/next of kin.

## Author Contributions

JV-R, JLS, HS, and IG conceived the study and drafted the manuscript. JV-R and SB performed the data curation and analysis. SB, JS, and FJ helped to draft the manuscript and contributed intellectually to the development of this paper. All authors reviewed and approved the final manuscript.

## Conflict of Interest

The authors declare that the research was conducted in the absence of any commercial or financial relationships that could be construed as a potential conflict of interest.

## Publisher's Note

All claims expressed in this article are solely those of the authors and do not necessarily represent those of their affiliated organizations, or those of the publisher, the editors and the reviewers. Any product that may be evaluated in this article, or claim that may be made by its manufacturer, is not guaranteed or endorsed by the publisher.
