# Supplementary material for: Negative Association Between Smoking and Positive SARS-CoV-2 Testing: Results From a Swiss Outpatient Sample Population
Source: Front Public Health. 2021 Nov 5;9:731981. doi: 10.3389/fpubh.2021.731981 (PMC8602063; doi:10.3389/fpubh.2021.731981)
Supplement: Supplementary file 1 [file Table_1.DOCX]

**Table S1** Population characteristics of the excluded sample (individuals living outside of the state of Geneva, without a valid RT-PCT result, and/or with missing data)

| **Population characteristics** | **Mean±SD or**  **Frequencies (%)** |
| --- | --- |
| N | 2,482 |
| Positive tests for SARS-CoV-2 | 395 (20%) |
| Smokers | 586 (24%) |
| Age (years) | 42.2±12.2 |
| Women | 1,596 (64%) |
| Healthcare workers | 1,550 (68%) |
| Household postal income (USD) | 142,332±30,998 |
| Respiratory diseases | 293 (12%) |
| Cardiovascular diseases and risk factors | 274 (11%) |
| Immunosuppressive conditions | 98 (4%) |
| Trip to a COVID-19 risk area | 236 (9%) |
| Contact with a SARS-CoV-2 positive individual | 1,302 (52%) |
