# Supplementary material for: Negative Association Between Smoking and Positive SARS-CoV-2 Testing: Results From a Swiss Outpatient Sample Population
Source: Front Public Health. 2021 Nov 5;9:731981. doi: 10.3389/fpubh.2021.731981 (PMC8602063; doi:10.3389/fpubh.2021.731981)
Supplement: Supplementary file 2 [file Table_2.DOCX]

**Table S2** Multiple logistic regression models for the association of smoking and testing positive for SARS-CoV-2. Analyses are adjusted for comorbidities, socioeconomic and demographic factors, and stratified by gender and non-healthcare population

|  | **Men**  **(n=2,076)** | | **Women**  **(n=3,093)** | | **Restricted to Non-healthcare population (n=3,146)** | |
| --- | --- | --- | --- | --- | --- | --- |
|  | **OR (95% CI)** | **p-value** | **OR (95% CI)** | **p-value** | **OR (95% CI)** | **p-value** |
| Smokers (yes vs no) | 0.43 (0.33, 0.56) | <0.001 | 0.44 (0.34, 0.57) | <0.001 | 0.41 (0.33, 0.51) | <0.001 |
| Age (years) | 1.01 (1.01, 1.02) | 0.001 | 0.99 (0.99, 1.00) | 0.42 | 1.01 (1.00, 1.01) | 0.004 |
| Women vs men | - | - | - | - | 0.75 (0.64, 0.89) | <0.001 |
| Healthcare workers vs general population | 0.54 (0.42, 0.69) | <0.001 | 0.66 (0.54, 0.79) | <0.001 | - | - |
| Household postal income (per 10,000 USD) | 0.99 (0.95, 1.02) | 0.40 | 0.96 (0.94, 0.99) | 0.01 | 0.97 (0.95, 1.00) | 0.07 |
| Respiratory diseases (yes vs no) | 0.77 (0.54, 1.06) | 0.12 | 0.63 (0.47, 0.83) | 0.001 | 0.71 (0.55, 0.92) | 0.01 |
| Cardiovascular diseases and risk factors (yes vs no) | 0.58 (0.41, 0.79) | <0.001 | 1.07 (0.80, 1.44) | 0.62 | 0.78 (0.60, 1.00) | 0.05 |
| Immunosuppressive conditions (yes vs no) | 0.42 (0.21, 0.77) | 0.008 | 0.80 (0.52, 1.19) | 0.28 | 0.60 (0.40, 0.86) | 0.007 |
| Trip to a COVID-19 risk area (yes vs no) | 1.49 (1.05, 2.08) | 0.02 | 1.19 (0.87, 1.61) | 0.25 | 1.29 (0.95, 1.74) | 0.09 |
| Contact with a SARS-CoV-2 positive individual (yes vs no) | 1.83 (1.47, 2.29) | <0.001 | 1.47 (1.22, 1.76) | <0.001 | 1.79 (1.50, 2.13) | <0.001 |
