# Supplementary material for: Negative Association Between Smoking and Positive SARS-CoV-2 Testing: Results From a Swiss Outpatient Sample Population
Source: Front Public Health. 2021 Nov 5;9:731981. doi: 10.3389/fpubh.2021.731981 (PMC8602063; doi:10.3389/fpubh.2021.731981)
Supplement: Supplementary file 3 [file Data_Sheet_1.pdf]

Hôpitaux Universitaires Genève	**Direction des soins / Département de Médecine Communautaire**

Formulaire pour la prise en soin d'un patient stable suspect d'une infection à SARS-CoV-2

COLLABORATEUR HUG/Collaborator HUG:
oui/yes ☐ non/no ☐
service/service: ______________
Si oui en contact avec patients/if yes, in contact with patients:
oui/yes ☐ non/no ☐

Site Ext hors HUG/site outside HUG:
oui/yes ☐ non/no ☐
Lieu/location: ______________________

Etiquette patient/ Patient label

NOM/Last name

PRENOM/Name

DATE DE NAISSANCE/date of birth

Tél/tel

Date/date:

Langue parlée/spoken language: français/french albanais/albanian anglais/english arabe/arabic espagnol/spanish farsi/farsi dari/dari portugais/portuguese roumain/romanian tigrinien/tigrinian autre/other:____________

Date de début des symptômes/date of symptoms onset :

A. **SYMPTÔMES/symptoms:**

Rhinorrhée/*Runny nose* **Odynodysphagie**/*sore throat* **Myalgies**/*muscular pain* **Frissons**/*chills*

Toux sèche/*dry cough* **Toux productive**/*productive cough* **Crachats rosés**/*pink sputum*

Fièvre (anamnestique ou objectivée)/*Fever* **Anosmie** (perte d'odorat)/*anosmia (loss of smell)*

Symptômes digestifs (nausées/diarrhées/douleurs abdo)/*abdominal symptoms* **asthénie** /*fatigue*

Dyspnée (peine à respirer)/*difficulty breathing* **Douleurs thoraciques**/ *thoracic pain*

céphalées/*headache*
Autre/other:

Allergies/allergies:

Traitements habituels/usual treatments:

B. CRITÈRES DE VULNÉRABILITÉ (facteurs de risque)/VULNERABILITY CRITERIA (risk factors):

Maladie respiratoire chronique/chronic respiratory disease:	oui/yes ☐	non/no ☐
Cardiopathie/heart disease:	oui/yes ☐	non/no ☐
ATCD d'infarctus/history of MI:	oui/yes ☐	non/no ☐
Immunosuppression/immunosuppression:	oui/yes ☐	non/no ☐
Greffé/ Transplanted:	oui/yes ☐	non/no ☐
Chimiothérapie récente/recent chemotherapy:	oui/yes ☐	non/no ☐
Cancer/cancer:	oui/yes ☐	non/no ☐
Diabète/diabetes:	oui/yes ☐	non/no ☐
Grossesse/pregnancy:	oui/yes ☐	non/no ☐
Patient prenant plus de 3 traitements médicamenteux/patient taking more than 3 drug treatments:	oui/yes ☐	non/no ☐
Tabagisme/smoking	oui/yes ☐	non/no ☐
HTA/hypertension	oui/yes ☐	non/no ☐

C. HABITUDES DE VIE/Lifestyle habits:

Prise récente (7 derniers jours) d'AINS (eg ibuprofen, diclofenac)/ recent (last 7 days) use of NSAIDs (eg ibuprofen, diclofenac):	oui/yes ☐	non/no ☐
Vaccin grippe (saison 2019-2020)/flu vaccine (2019-2020 season):	oui/yes ☐	non/no ☐
Patient SDF/homeless patient:	oui/yes ☐	non/no ☐
Foyer (migrants/réfugiés)/shelter (migrants/refugees):	oui/yes ☐	non/no ☐
Résident EMS/resident nursing home:	oui/yes ☐	non/no ☐
Environnement psycho-social difficile/difficult psycho-social environment:	oui/yes ☐	non/no ☐

Autre structure de vie collective/other group living facilities: _____________

Nombre de personnes vivant dans le même appartement/foyer/number of people living in the same apartment/shelter: ____

D. EXPOSITION (lien épidémiologique)/ EXPOSURE (epidemiological link):

1. Avez-vous voyagé dans les 14 derniers jours ? **oui/yes ☐** **non/no ☐**

Have you travelled in the last 14 days?

Si oui (if yes) :

Pays/country:_____________, ville/city:_________________Nombre de jour/number of days: _____________

Pays/country:_____________, ville/city:_________________Nombre de jour/number of days: _ _____________

Quelles activités sociales ou professionnelles avez-vous faites dans ces pays/What social or professional activities have you done in these countries?

2. Avez-vous été en contact avec une personne confirmée positive au SARS-CoV-2 dans les 14 derniers jours ?

Have you been in close contact with a SARS-CoV-2 confirmed patient in the last 14 days?

oui/yes ☐ **non/no ☐**

Lieu/place: Date/date:

3. Etiez-vous en quarantaine en tant que contact proche d'un cas confirmé SARS-CoV-2 ?

Were you in quarantine due to a close contact of a confirmed SARS-CoV-2 case?

oui/yes ☐ **non/no ☐**

FROTTIS/smear: **oui/yes ☐** **non/no ☐**

Veuillez préciser si frottis non effectué/Please specify if the smear was not performed:_________________
